# Handgrip Strength, Depression, Dementia, Cognitive Function, and Their Predictive Effect on Functional Independence in Older Adults

**DOI:** 10.3390/medicina61061030

**Published:** 2025-06-01

**Authors:** Juan Antonio Campos-Gutiérrez, Enrique Diaz De León-González, Hugo Gutiérrez Hermosillo, Ricardo M. Cerda, Georgina Mayela Núñez Rocha, Jorge Zamarripa, Ricardo López-García, Guillermo Cano-Verdugo, Rocío Martínez-Hernández

**Affiliations:** 1 Escuela de Medicina y Ciencias de la Salud, Tecnologico de Monterrey, Monterrey 64700, Mexico; 2 Casa de Reposo Santa Teresa, San Nicolás de los Garza 66400, Mexico; ricardocerda_@yahoo.com.mx (R.M.C.); rocio.martinezhe@uanl.edu.mx (R.M.-H.); 3 Departamento de Geriatría, UMAE 21 Instituto Mexicano del Seguro Social, Monterrey 64000, Mexico; edleon20@hotmail.com; 4 Departamento de Geriatría, Hospital Aranda de la Parra, Guanajuato 37000, Mexico; hugocus@hotmail.com; 5 Escuela Nacional de Estudios Superiores, Universidad Nacional Autónoma de México, León Guanajuato 37684, Mexico; 6 Facultad de Salud Pública y Nutrición, Universidad Autónoma de Nuevo León, Monterrey 66455, Mexico; georgina.nunezrc@uanl.edu.mx (G.M.N.R.); cavgod0002@uanl.edu.mx (G.C.-V.); 7 Facultad de Organización Deportiva, Universidad Autónoma de Nuevo León, San Nicolas de los Garza 66455, Mexico; jorge.zamarriparv@uanl.edu.mx (J.Z.); ricardo.lopezgr@uanl.edu.mx (R.L.-G.)

**Keywords:** handgrip strength, depression, dementia, cognitive level, independence, predictive effect

## Abstract

*Background and Objectives*: Globally, there is a demographic transition toward an increase in the number of older adults, and with it, the comorbidities associated with aging. This requires healthcare providers to understand which variables can affect functional independence for performing activities of daily living. The general objective of this study was to determine the predictive effect of left and right handgrip strength, depression, mild to moderate dementia, and cognitive function on functional independence in older adults. *Materials and Methods*: This study featured a predictive cross-sectional design with *n* = 84 older adults with some level of physical independence; older adults with completely limited physical independence and those with severe dementia were excluded. To assess depression, the Geriatric Depression Scale was used; for dementia, the Hachinski Ischemic Scale was used; for cognitive impairment, the Folstein version of the MMSE was used; for functional independence, the Barthel Index was used; for handgrip, a 90-kg Dynatron^®^ professional hydraulic dynamometer (UT 84121) Number Series. 11010141, from the Dynatronics Corporation located in Salt Lake City, Utah USA, was used. *Results*: In total, 58.8% of the participants were female, with a mean age of 84.89 ± 7.095, with ranges from 68 to 102 years. Multiple regression analysis showed that the level of cognition, left-hand grip strength, and a low level of depression are strong predictors of independence in activities of daily living in the elderly, with an explained variance of R^2^ = 0.34. *Conclusions*: Cognitive function, left-hand grip strength, and depression significantly predict the independence of older adults. Studies with larger sample sizes are recommended to confirm the veracity of the results and to design methodologically rigorous interventions that include psychological aspects such as cognitive stimulation, promoting physical activity, and addressing depressive problems to improve the functional independence of older adults.

## 1. Introduction

The World Health Organization (WHO) reports that the older adult population has increased considerably in recent years [1,2]. This population curve transition is occurring globally and nationally [National Institute of Statistics and Geography (INEGI), 2018] and is expected to continue rising [3,4]. This demographic landscape presents a pressing challenge for public health systems and the global economy. Therefore, knowing and understanding the health of older adults become a necessity [1,5,6,7,8], since during this stage of life, the body undergoes important physiological changes, especially the musculoskeletal system, such as the decrease in muscle mass and quality, which increases the risk of falls, bone fractures, hospitalizations, mortality, and deterioration of functional capacity, thus decreasing the quality of life of older adults and their caregivers [6,9,10,11].

Within geriatrics and gerontology, the assessment of functional capacity or independence in older adults is an indicator of health and quality of life [1,9,12,13,14]. Currently, functional capacity in older adults is a way of understanding and recognizing their functioning and disability. It analyzes the level of performance of daily activities and the development of social situations. It is determined by characteristics of the social environment, genetic inheritance, and modifiable lifestyles. Therefore, its study in older adults is relevant for improving health levels and quality of life [13,15,16].

Some research studies indicate that functional capacity is determined by muscle strength, which refers to the maximum force of skeletal muscle voluntarily infringed with short duration [17], and add that a low level of muscle strength is associated with multiple health problems, including disability, chronic morbidities, and premature mortality [14,18,19]; muscle strength is also considered a biomarker of general health status and an indicator of health in middle-aged and older adults that could be used to identify possible health risks [11,17,18,20].

Other studies indicate that depression, cognitive impairment, or cognitive decline (reduction in reasoning, memory, and understanding abilities) are clinical conditions that decrease independence in carrying out daily activities. In turn, these conditions increase the risk of suffering from chronic diseases and the risk of mortality [8,16,21]. Also, it is mentioned that older adults with cognitive impairment develop depression as a reaction to the presence of cognitive decline and the loss of independence to carry out activities of daily living [8], and cognitive impairment, dementia, and depression increase the risk of suffering from diseases such as Alzheimer’s and, consequently, functional decline [7,22,23]. Likewise, they add that dementia is the main predictor of the functional independence of the elderly [24].

However, there is no consensus on which variables have the greatest predictive effect on the functional independence of the elderly, which is why the present research study is designed. This study has a general objective to determine the predictive effect that the grip strength of the left hand, the right hand, depression, dementia, and cognitive functionality have on the functional independence of the elderly. Particularly, this study aims to establish the difference between the grip strength of the left hand, the right hand, depression, dementia, and cognitive functionality among older adults with different levels of independence, with the aim of designing interventions that help maintain and/or improve the functional independence of the elderly.

## 2. Materials and Methods

This is a quantitative, non-experimental study with a cross-sectional and predictive design, seeking to determine which independent variable has the greatest predictive effect on the dependent variable [25].

### 2.1. Participants and Sample

The study population consisted of 96 older adults residing in a nursing home in Nuevo León, Mexico. The sample was selected non-probabilistically for convenience [26]. Twelve surveys were eliminated due to incomplete information, leaving a final sample of 84 older adults.

Among the selection criteria, older adults with some level of physical independence (who could eat alone and ambulate with assistance or alone) were considered; older adults with completely limited physical independence were excluded, that is, those permanently bedridden and who required assistance with eating and bathing. Older adults with advanced dementia were also excluded, and incomplete surveys were eliminated.

### 2.2. Measurement Variables

All participants were interviewed by a physician trained for this purpose. To collect sociodemographic data, an ad hoc questionnaire was designed containing questions about the institution of medical affiliation, sex, age, marital status, and diagnosed pathologies.

To assess the level of functional independence, the Barthel Index was used. This is a scale used to measure the level of daily independence, assessing an individual’s ability to perform activities independently. It consists of 10 questions that inquire about daily activities and has an ordinal measurement level. Each item is scored with three possible responses: 0 if dependent, 5 if assisted, and 10 if independent. The score ranges from 0 to 100; the higher the score, the greater the level of independence. The suggested cutoff points are as follows: 0–20 = total dependence; 21–60 = severe dependence; 61–90 = moderate dependence; 91–99 = low dependency; and 100 = independence [5,12,27].

To assess depression levels, the Geriatric Depression Scale (GDS) by Yesavage et al. [28] was used. This scale measures depression in healthy, ill, or elderly adults with mild to moderate cognitive impairment in different settings (hospital, long-term care, and community). It consists of 15 questions with dichotomous responses (Yes = 1, No = 0). For final interpretation, one point is awarded for each response (a higher score indicates a greater presence of depressive symptoms). It takes 5 to 7 min to administer. The cutoff points are as follows: 0–4 points indicate no depressive symptoms; 5–8 points indicate the presence of mild depressive symptoms; 9–10 points indicate the presence of moderate depressive symptoms; and 12–15 points indicate the presence of severe depressive symptoms [28,29].

The Hachinski Ischemic Scale (HIS) was used to measure the main types of dementia (primary degenerative, vascular or multi-infarct, and mixed). It contains a series of questions with clinical characteristics that are scored to obtain a number indicating the probability of vascular dementia (VD). These are clinical conditions with a hemorrhagic, ischemic, or ischemic/hypoxic origin, with a typically abrupt onset and progressive deterioration, with somatic complaints and some emotional incontinence. Scores below 4 suggest neurodegenerative disorders. A score of 7 or more indicates a higher likelihood of vascular dementia, and a score of 4 or less indicates a higher likelihood of Alzheimer’s dementia [30].

To assess cognitive level, a version of Folstein’s Mini-Mental State Examination (MMSE) was used. Its characteristics are simple and brief. It consists of 11 items and examines various cognitive functions: temporal–spatial orientation, fixation and short-term memory, attention, language, and visual–constructive skills. Higher scores indicate greater cognitive functioning; the maximum score is 30 points [31].

Grip strength was measured by the same trained physician using a Dynatron^®^ 90 kg professional hydraulic dynamometer (UT 84121) Number Series. 11010141, from the Dynatronics Corporation located in Salt Lake City, UT, USA, which is used to determine upper body isometric strength. The test was performed with the patient’s elbow at 90° of flexion, encouraging maximum strength. Three measurements were taken on each hand, allowing one minute of rest between tests to ensure muscle recovery, and finally, the average of the three measurements was recorded [32].

This study was conducted in accordance with the Declaration of Helsinki [33] and the General Health Law on Research [34] and was therefore approved by the Research Ethics Committee.

### 2.3. Statistical Analysis

Descriptive statistics were performed, measures of central tendency were used for continuous variables (mean and standard deviation), and frequencies and percentages were used for categorized variables.

To meet the specific objective of establishing the difference between left-hand and right-hand grip strength, depression, dementia, and cognitive function among older adults with different levels of independence, the data set was first determined to be normally distributed using the Kolmogorov–Smirnov test. Data were observed to be non-normally distributed on three scales (depression, dementia, and weight). The data were then transformed using the logarithmic method. Once transformed, *Student’s t* test for independent samples was used. *p* values < 0.05 were considered statistically significant.

To meet the overall objective of understanding the predictive effect of left-hand and right-hand grip strength, depression, dementia, and cognitive function on functional independence in older adults, a multiple linear regression analysis was performed using a stepwise method. The dependent variable was the level of independence, and the independent variables were right and left-hand grip strength, depression, dementia, cognitive function, and weight. *p* values < 0.05 were considered statistically significant.

Statistical analyses were performed using IBM Statistical Package for the Social Sciences (SPSS, v. 27) for Windows. Effect size and statistical power were analyzed using G*Power version 3.1.9.7.

## 3. Results

The majority of participants were female (58.8%), with a mean age of 84.89 ± 7.095. A total of 65.9% of participants were affiliated with the Mexican Social Security Institute (IMSS), 14.1% belonged to the Institute of Security and Social Services for State Workers (ISSSTE), and only 8.2% were enrolled in public insurance. Most participants reported being widowed (51.8%), 24.7% were in a common-law marriage, 14.1% were single, and only 9.4% were divorced.

Of the total participants, 42.4% wore diapers, 16.5% had diabetes mellitus, 44.7% had hypertension, 8.2% had a history of cancer, 20.0% had chronic obstructive pulmonary disease (COPD), 1.2% had some type of heart disease, 9.4% had rheumatism, 8.2% had chronic kidney failure, and 11.8% reported having had a fall. Statistically significant differences (*p* = 0.02) were observed in the prevalence of chronic obstructive pulmonary disease by sex, with the proportion being higher (28%) in women than in men (8.6%) (see Table 1).

Regarding the level of independence, a mean score of 57.61 ± 34.11 was obtained, ranging from 1 to 100 points, indicating moderate dependence in the total population. The results revealed that 70.6% of the participants had severe dependence, meaning they had very limited mobility and could only perform certain activities with the help of another person.

Analysis of Student’s t test for independent samples showed greater levels of grip strength in the left and right hands in people with moderate dependence compared to those with severe dependence, with statistically significant differences (*p* = 0.01 and *p* = 0.02). Statistically significant differences (*p* = 0.001) were also found in the level of cognitive function, with people with moderate dependence having higher levels of cognitive function compared to those with severe dependence (see Table 2).

The multiple linear regression analysis showed that, of the six variables analyzed (grip strength of the left and right hand, level of depression, level of dementia, cognitive functions, and weight), only the level of cognition, the grip strength of the left hand, and a lower level of depression are predictors of independence in daily activities of the elderly with an explained variance of *R*^2^ = 0.34, which means that the defined model on independence explains 34% of the variance of the dependent variable, which is statistically significant (*p* = 0.001), with 1-*β* 0.90 (see Table 3).

## 4. Discussion

This main objective of this study was to determine the predictive effect of left and right-hand grip strength, depression, mild to moderate dementia, and cognitive function on functional independence in older adults in a nursing home, being one of the few studies carried out in Mexico that addresses a population of older adults who are recruited in a nursing home and that seeks to establish which variables should be worked on to prevent the loss of functionality in older adults, considering variables that affect older adults but are often forgotten because they influence mood more, such as depression.

Regarding the level of independence, the mean score for the total population was 57.61 ± 34.11 with a range of 1 to 100 points, which translates to a moderate level of independence. It was also observed that 70.6% of the total participants had severe dependency. These results are consistent with those reported by other researchers, where the prevalence of older adults with limited functional capacity is high [35], and differ from those reported by Luna-Orozco et al. [22], who reported that a low percentage of older adults have limitations in performing activities of daily living. The concordant results could be explained by the fact that the data were collected in a nursing home, so it is expected that most participants would have partial or very severe limitations in independently performing daily activities. The discrepant results could be due to the fact that in the study by Luna-Orozco et al. [22], the participants were older adults from the general population; These results show that it is important to design interventions that help maintain physical functionality in older adults; this is because when their functionality in daily activities substantially decrease, they tend to be sheltered in nursing homes or asylums, with the aim of taking care of their well-being due to lacking the independence that would allow them to care for themselves.

Regarding the particular objective, it was observed that there are statistically significant differences in the grip strength of the left hand (15.37 ± 5.8; 19.46 ± 7.27; *p* = 0.01) and right hand (16.78 ± 5.51; 20.02 ± 7.40; *p* = 0.02) between older adults with moderate dependence versus those with severe dependence, with older adults with moderate dependence having greater grip strength in the left and right hands. Statistically significant differences (*p* = 0.01) were also observed in the cognitive level, with adults with moderate dependence having a higher cognitive level (24.00 ± 5.79) compared to adults with severe dependence (18.17 ± 7.0).

These results agree with those reported by other researchers where handgrip strength is greater in adults with a higher level of independence compared to those with severe impairment of functional independence [4,35]; adding to the above, it is important to mention that in other studies [14,18,19], it has been found that a low level of handgrip strength is associated with multiple health problems, including disability, chronic morbidities, sarcopenia, vascular accidents, and premature mortality, among others. These results highlight the importance of promoting active lifestyles in older adults in order to contribute to preserving physical independence and improving quality of life.

Finally, to meet the general objective, it was found that the level of cognition, the grip strength of the left hand, and a lower level of depression are strong predictors of older adults’ independence in daily activities, with an explained variance of *R*^2^ = 0.34, which means that the defined model on independence explains 34% of the variance of the dependent variable; significantly (*p* = 0.001), these results agree with those reported by other researchers [7,22,36], where it is mentioned that cognition, old age, and depression are strong predictors of independence to carry out activities of daily living. Likewise, they partially agree with what has been reported in other studies, where an association was found between the likelihood of dementia, physical activity, depression, and limitations in independence of daily living [16,23]; in another study, it was found that grip strength and cognitive status, and grip strength and frailty, had a positive relationship, and that depression and cognitive status were closely related to the physical frailty of older adults [4,22].

As can be seen, the results of this study align with the findings of other researchers, who expose the importance of cognitive functioning, hand grip strength, and depression in independence for performing activities of daily living in older adults in a nursing home, and add two findings that are noteworthy. The first is the predictive effect that the left hand has on the functionality of older adults; however, these results have already been observed in other studies [37,38], where the left hand has a direct relationship with mortality, some vascular diseases, and the frailty of older adults. These results can be explained by the fact that in some pathologies, the small distal muscles are affected before the large proximal muscles, and by the difference (5–10 mm of mercury) between the blood pressure of both arms, which in turn could influence cognitive functioning that directly affects the functionality of older adults. It can also be explained by the fact that the majority of participants were right-handed. However, it is important to view these results with caution, firstly, because the arm dominance of the participants is unknown, and secondly, because blood pressure, arm dominance, and the presence of pathologies such as uremia were not considered as moderating or adjustment variables.

The second valuable finding is the level of depression, which should be given more attention during adulthood, since during this stage of life, older adults face potential factors (existence of chronic diseases, a perception of poor health status, reduction in interaction or social role, and abandonment, among others) that can trigger significant depressive clinical symptoms that substantially affect independence in activities of daily living, and more attention should be paid to older adults who are sheltered in homes or specialized care clinics for older adults; in parallel, cognitive stimulation, the promotion of physical activity, and mental healthcare during adult life should be given greater attention in routine clinical practice to promote healthy and quality aging.

## 5. Conclusions

The findings of this study show that the main predictor of independence in older adults is cognitive function, followed by left-hand grip strength and depression. This study has great clinical relevance, since most medical interventions focus on treating comorbidities through medication, and interventions based on promoting physical activity and psychological aspects, such as cognitive stimulation and the management of depressive disorders in older adults, are more limited. Therefore, it would be important to address cognitive decline as a first line of action, since cognitive losses, coupled with a level of general physical strength, can create a series of events that further affect health and, consequently, the functionality and independence of older adults. It is also important to mention that depressive disorders can trigger a decline in cognition. It is suggested that the sample size be increased and that studies with an experimental approach be designed, in which physical therapy and cognitive stimulation are provided and the effect of the intervention is evaluated.

## 6. Limitations

Important limitations worth mentioning include the minimum sample size required for this type of study and the statistical power (*1-β*) = 0.80 used. Other important limitations worth highlighting include the potential bias of considering older adults who were able to eat and move around alone, or with the support of someone else, and considering older adults from a single nursing home. Additionally, there was no assessment of other variables that may be involved in functional independence, such as gait assessment. Other limitations include the lack of information on the length of time the older adults were in the nursing home, information on their dominant and non-dominant hand, nutritional status, the moderating effect or statistical adjustment with other variables that could influence the independence of the older adult, such as sex, blood pressure, arm dominance, and the presence of pathologies such as uremia.

Therefore, the sample size of future studies should be increased, *1-β*, and studies should be conducted on a non-captive population, analyzing comparisons between the open population and the captive population (people who are in nursing homes, asylums. or assistance clinics for the elderly) and considering more variables that could influence functional independence, such as added comorbidities, nutritional status, level and duration of depression, physical activity, development or habits of social interaction, and arm dominance, among others.

## Figures and Tables

**Table 1:** Prevalence of comorbidities in older adults (*n* = 84 *).

Diseases		Gender		Total	*p*	V for Cramer
		Men	Women			
Diaper use	Yes	34.3%	48.0%	42.4%	0.20	0.13
	No	65.7%	52%	57.6%		
Diabetes mellitus	Yes	25.7%	10.0%	16.5%	0.05	0.20
	No	74.3%	90%	83.5%		
Hypertension	Yes	48.6%	42.0%	44.7%	0.54	0.06
	No	51.4%	58.0%	55.3%		
History of cancer	Yes	8.6%	8%	8.2%	0.92	0.01
	No	91.4%	92.0%	91.8%		
Chronic obstructive pulmonary disease	Yes	8.6%	28%	20.0%	0.02	0.23
	No	91.4%	72%	80.0%		
Heart disease	Yes	2.9%	0%	1.2%	0.22	0.13
	No	97.1%	100%	98.8%		
Rheumatism	Yes	5.7	12	9.4	0.32	0.10
	No	94.3	88	90.6		
Chronic kidney failure	Yes	8.6	8	8.2	0.92	0.01
	No	91.4	92	91.8		
Previous falls	Yes	14.3	10	11.8	0.54	0.06
	No	85.7	90	88.2		

Note: Prepared by the authors; * sample number; *p* < 0.05 were statistically significant.

**Table 2:** Differences in left-hand grip strength, right-hand grip strength, depression level, dementia level, cognitive function, and weight by severe and moderate dependence (*n* = 84 *).

Variables	Severe Dependence		Moderate Dependence		*p*	*1-β*
	M ± DS	IC 95%	M ± DS	IC 95%		
Left-Hand Grip Strength	15.37 ± 5.8	13.85;16.92	19.46 ± 7.27	16.38;22.20	0.01	0.54
Right-Hand Grip Strength	16.78 ± 5.51	15.41;18.14	20.02 ± 7.40	17.26;22.82	0.02	0.40
Level of Depression	4.62 ± 2.44	4.00;5.25	3.60 ± 2.61	2.69;4.65	0.09	0.54
Level of Dementia	1.52 ± 0.725	1.34;1.71	1.48 ± 0.653	1.24;1.76	0.82	0.10
Cognitive Functions	18.17 ± 7.0	16.36;19.90	24.00 ± 5.79	21.61;26.13	0.01	0.90
Weight	57.36 ± 13.1	54.16;60.79	58.34 ± 14.07	53.07;63.47	0.76	0.54

Note: Prepared by the authors; * sample number; *p* < 0.05 were statistically significant.

**Table 3:** Multiple linear regression analysis, where the independent variables are the level of left- and right-hand grip strength, level of depression, level of dementia, cognitive functions, and weight, and the dependent variable is the level of independence (*n* = 84 *).

Predictor Variables	*B*	SE B	Beta Coefficient	*R* ^2^	F *(gl)*	*p*	*1-β*
Constant	7.828	12.034		0.34	14.477 (3.81)	0.001	0.90
Cognitive Functions	1.740	0.444	0.367				
Left-Hand Grip Strength	1.630	0.489	0.312				
Level of Depression	−2.740	1.215	−0.203				

Note: *B* = unstandardized coefficients; SE B = Standard error; Beta = Standardized; *R*^2^ = coefficient of determination; F *(df)* = F value with the regression degree of freedom value; *p* < 0.05 was considered statistically significant; *1-β* = statistical power. * sample number.

## Data Availability

The data supporting the findings of this study are available from the corresponding author, J.A.C.-G., upon request and with reasonable justification.

## References

[1] Echeverría A., Astorga C., Fernández C., Salgado M., Villalobos Dintrans P. (2022). Funcionalidad y Personas Mayores: ¿dónde Estamos y Hacia Dónde Ir?. Rev. Panam. Salud Pública.

[2] (2021). OMS Informe Sobre La Situación Mundial de La Respuesta de La Salud Pública a La Demencia: Resumen Ejecutivo [Global Status Report on the Public Health Response to Dementia: Executive Summary].

[3] INEGI (2018). Encuesta de Evaluación Cognitiva Para La ENASEM 2021.

[4] Vancea Nemirschi A.T., Lupu A.A., Aivaz K.-A., Iliescu M.G., Deriaz M., Marzan M., Spiru L. (2024). Exploring the Connections Between Grip Strength, Nutritional Status, Frailty, Depression, and Cognition as Initial Assessment Tools in Geriatric Rehabilitation—A Pilot Study. Medicina.

[5] Duarte-Ayala R.E., Velasco-Rojano Á.E. (2021). Validación Psicométrica Del Índice de Barthel En Adultos Mayores Mexicanos. Horiz. Sanit..

[6] Mancilla S.E., Ramos F.S., Morales B.P. (2016). Fuerza de Prensión Manual Según Edad, Género y Condición Funcional En Adultos Mayores Chilenos Entre 60 y 91 Años. Rev. Med. Chil..

[7] Pérez A., Oviedo D., Britton G. (2018). Deterioro Cognitivo Leve y Depresión En El Adulto Mayor. Investig. Y Pensam. Crítico.

[8] Rodríguez-Vargas M., Rojas-Pupo L., Pérez-Solís D., Marrero-Pérez Y., Gallardo-Morales I., Durán-Cordovés L. (2021). Cognitive Functioning of Older Adults with Depression. Rev. Arch. Médico Camagüey.

[9] Cabañero-Martínez M.J., Cabrero-García J., Richart-Martínez M., Muñoz-Mendoza C.L. (2009). The Spanish Versions of the Barthel Index (BI) and the Katz Index (KI) of Activities of Daily Living (ADL): A Structured Review. Arch. Gerontol. Geriatr..

[10] Lawman H.G., Troiano R.P., Perna F.M., Wang C.-Y., Fryar C.D., Ogden C.L. (2016). Associations of Relative Handgrip Strength and Cardiovascular Disease Biomarkers in U.S. Adults, 2011–2012. Am. J. Prev. Med..

[11] Porto J.M., Nakaishi A.P.M., Cangussu-Oliveira L.M., Freire Júnior R.C., Spilla S.B., Abreu D.C.C. (2019). de Relationship between Grip Strength and Global Muscle Strength in Community-Dwelling Older People. Arch. Gerontol. Geriatr..

[12] Bernaola-Sagardui I. (2018). Validación Del Índice de Barthel En La Población Española. Enferm. Clin..

[13] Rendón-Torres L., Sierra-Rojas I., Benavides-Guerrero C., Botello-Moreno Y., Guajardo-Balderas V., García-Perales L. (2021). Factores Predictores Del Deterioro Cognitivo En Personas Mayores de 60 Años. Enferm. Clin..

[14] Russo M.J., Kañevsky A., Leis A., Iturry M., Roncoroni M., Serrano C., Cristalli D., Ure J., Zuin D. (2020). Role of Physical Activity in Preventing Cognitive Impairment and Dementia in Older Adults: A Systematic Review. Neurol. Argent..

[15] Martín-Aranda R. (2018). Physical Activity and Quality of Life in the Elderly. A Narrative Review. Rev. Habanera Cienc. Médicas.

[16] Rist P.M., Capistrant B.D., Wu Q., Marden J.R., Glymour M.M. (2014). Dementia and Dependence. Neurology.

[17] Bohannon R.W. (2015). Muscle Strength. Clinical and Prognostic Value of Hand-Grip Dynamometry. Curr. Opin. Clin. Nutr. Metab. Care.

[18] McGrath R.P., Kraemer W.J., Snih S.A., Peterson M.D. (2018). Handgrip Strength and Health in Aging Adults. Sports Med..

[19] Wang Y.-C., Bohannon R.W., Li X., Sindhu B., Kapellusch J. (2018). Hand-Grip Strength: Normative Reference Values and Equations for Individuals 18 to 85 Years of Age Residing in the United States. J. Orthop. Sports Phys. Ther..

[20] Bohannon R.W. (2019). Grip Strength: An Indispensable Biomarker For Older Adults. Clin. Interv. Aging.

[21] Lövdén M., Fratiglioni L., Glymour M.M., Lindenberger U., Tucker-Drob E.M. (2020). Education and Cognitive Functioning Across the Life Span. Psychol. Sci. Public. Interestig..

[22] Luna-Orozco K., Fernández-Niño J.A., Astudillo-García C.I. (2020). Asociación Entre La Discapacidad Física y La Incidencia de Síntomas Depresivos En Adultos Mayores Mexicanos. Biomédica.

[23] Zasadzka E., Pieczyńska A., Trzmiel T., Kleka P., Pawlaczyk M. (2021). Correlation between Handgrip Strength and Depression in Older Adults—A Systematic Review and a Meta-Analysis. Int. J. Environ. Res. Public Health.

[24] Harwood R.H., Goldberg S.E., Brand A., van Der Wardt V., Booth V., Di Lorito C., Hoare Z., Hancox J., Bajwa R., Burgon C. (2023). Promoting Activity, Independence, and Stability in Early Dementia and Mild Cognitive Impairment (PrAISED): Randomised Controlled Trial. BMJ.

[25] Vilà Baños R., Torrado Fonseca M., Torrado Fonseca M. (2019). Análisis de Regresión Lineal Múltiple Con SPSS: Un Ejemplo Práctico. Rev. D’innovació I Recer. En. Educ..

[26] Hernández-Sampieri R., Mendoza C., McGrawHill (2018). Metodología de La Investigación: Las Rutas Cuantitativa, Cualitativa y Mixta.

[27] Cid-Ruzafa J., Damián-Moreno J. (1997). Valoración de La Discapacidad Física: El Índice de Barthel. Rev. Esp. Salud Pública.

[28] Yesavage J.A., Brink T.L., Rose T.L., Lum O., Huang V., Adey M., Leirer V.O. (1982). Development and Validation of a Geriatric Depression Screening Scale: A Preliminary Report. J. Psychiatr. Res..

[29] Baker F.M., Espino D.V. (1997). A Spanish Version of the Geriatric Depression Scale in Mexican-American Elders. Int. J. Geriatr. Psychiatry.

[30] Molina D.M. (2016). The Role of Neuropsychological Assessment in the Diagnosis and Monitoring of Dementia. Rev. Médica Clínica Las. Condes.

[31] Escribano-Aparicio M.V., Pérez-Dively F.J., García-García A., Pérez-Martín L., Romero G., Ferrer E., Martín-Correa M. (1999). Validación Del MMSE de Folstein En Una Población Española de Bajo Nivel Educativo1. Hosp. Geriátrico.

[32] Gutiérrez-Hermosillo H., de León-González E.D., Medina-Chávez J.H., Torres-Naranjo F., Martínez-Cordero C., Ferrari S. (2020). Hand Grip Strength and Early Mortality after Hip Fracture. Arch. Osteoporos..

[33] Asociación Médica Mundial Declaración de Helsinki de La AMM-Principios Éticos Para Las Investigaciones Médicas En Seres Humanos. https://www.guatemala-cds.com/wp-content/uploads/2020/08/Declaración-de-Helsinki-de-la-AMM-Principios-éticos-para-las-investigaciones-médicas-en-seres-humanos.pdf.

[34] Diario Oficial de la Federación Reglamento de La Ley General de Salud En Materia de Investigación Para La Salud. https://www.gob.mx/insabi/prensa/reglamentos-de-la-ley-general-de-salud-275018?idiom=es.

[35] Tarducci G., Gárgano S., Paganini A., Vidueiros S., Gandini A., Fernández I., Nápoli C., Pallaro A. (2020). Condición Física Saludable y Su Relación Con Habilidades Básicas Para La Independencia Del Adulto Mayor. Hacia Promoción Salud.

[36] Benke T., Delazer M., Sanin G., Schmidt H., Seiler S., Ransmayr G., Dal-Bianco P., Uranüs M., Marksteiner J., Leblhuber F. (2013). Cognition, Gender, and Functional Abilities in Alzheimer’s Disease: How Are They Related?. J. Alzheimer’s Dis..

[37] Clark C.E., Taylor R., Shore A.C., Ukoumunne O.C., Campbell J.L. (2012). Association of a Difference in Systolic Blood Pressure between Arms with Vascular Disease and Mortality: A Systematic Review and Meta-Analysis. Lancet.

[38] Hellberg M., Wiberg E.M., Simonsen O., Höglund P., Clyne N. (2014). Small Distal Muscles and Balance Predict Survival in End-Stage Renal Disease. Nephron Clin. Pract..

